# Therapeutic Potential of Extracellular Vesicles

**DOI:** 10.3389/fimmu.2014.00658

**Published:** 2014-12-19

**Authors:** Ana Maria Merino, Martin Johannes Hoogduijn, Francesc E. Borras, Marcella Franquesa

**Affiliations:** ^1^Bellvitge Biomedical Research Institute (IDIBELL), Barcelona, Spain; ^2^Erasmus University Medical Center, Rotterdam, Netherlands; ^3^Institute of the Germans Trias i Pujol Foundation (IGTP), Barcelona, Spain

**Keywords:** extracellular vesicles, exosomes, tetraspanins, immunotherapy, tumor growth, regenerative medicine

Extracellular vesicles (EV) have emerged as important mediators of intercellular communication. By their origin, we can find vesicles derived from plasma membrane such as microvesicles, ectosomes, and membrane particles or exosomes, which originate in the endosomal membrane compartment. They contain numerous proteins, lipids, and even nucleic acids like mRNA and miRNA that can affect the cells that encounter these structures in complex ways.

The EV have recently gained interest for their therapeutic potential both as a treatment itself and as a biomarker of several pathologies. The research lines involving EV cover a wide range of aspects from basic research on the EV biology to the manipulation or monitoring of EV for therapeutic purposes.

In this Research Topic, Andreu and Yáñez-Mó (1) discuss the evidence for proteins of the tetraspanin family to be considered as exosome markers and deliberate on their functions on exosomes, starting from biogenesis and cargo selection to the final binding and fusion with the target cell. They also review the ability of exosomes of presenting antigen and the role of tetraspanins in this process. Moreno-Gonzalo et al. (2) focus their review on the suggestion that the incorporation of cargo into EV is a regulated process. They describe the post-translational modification found in exosomal protein and their role in the complex mechanism of cargo sorting into the vesicles. In a perspective article, Baixauli et al. (3) discuss about the function of exosomes as a means to alleviate intracellular stress conditions, and how secretion of harmful or unwanted material in exosomes, in coordination with the autophagy-lysosomal pathway, is essential to preserve intracellular protein and RNA homeostasis. They also give an overview about the consequences of the spreading of exosomal content in physiological and pathological situations and strategies for therapeutic intervention. Compared with other secreted vesicles, exosomes have much better defined biophysical and biochemical properties. However, the isolation and characterization protocols are not homogeneous in the published articles. Franquesa et al. (4) contribute to this research topic with a methods article in which they propose new controls for the detection and quantification of EV derived from mesenchymal stem cells. These three reviews and the method article provide an overview about the most important basic concepts in the EV field.

Immunomodulatory potential is one of the main focuses of study regarding EV that captures the attention of researchers working in several fields of medicine. Zhang et al. (5) review studies which demonstrate the immense immunotherapeutic potential of EV by effects on both immune and non-immune cells and also highlight the divergent effects of EV on the immune system. They argue that this divergence could be due to the cell source of EV and other parameters such as the classes of EV and the methods of isolation. Blázquez et al. (6) contribute to this topic with an original article in which they describe *in vitro* experiments aimed at characterizing the immunomodulatory effect of EV derived from mesenchymal stem cells on stimulated T cells. The recent description of parasite EV in protozoan and helminths suggests that EV can play an important role in host–parasite communication by immunomodulation of the host immune response against the parasite. Montaner et al. (7) review current clinical trials about the use of helminth secretory products to treat chronic inflammatory and autoimmune diseases. Interestingly, some of the characterized parasitic immunomodulatory proteins have been identified in EV, and they discuss the intriguing possibility of the therapeutic use of parasitic EV.

The therapeutic potential of EV is studied in different fields such as organ transplantation, HIV, cardiovascular diseases (CVs), tumor biology, and regenerative medicine. In this research topic, nine reviews overview the role of EV in several pathologies. Two reviews describe the role of EV derived from immune cells in the transplantation field with different viewpoints. Agarwal et al. (8) summarize the EV contribution to immune regulation and their therapeutic potential in preventing graft rejection and Monguió-Tortajada et al. (9) discuss the mechanisms involved in organ tolerance mediated by the administration of EV. In the HIV field, Soares (10) review the relevance of CD4 vesicles exocytosis to immune regulation and to HIV-1 pathogenesis. CV continues to be a leading cause of morbidity and mortality worldwide. EV are considerably elevated in CV associated with inflammation and have also been shown to have proangiogenic and cardioprotective properties. Fleury et al. (11) provide an update of cellular processes modulated by EV of specific interest for the treatment of cardiovascular pathologies with special focus on morphogen sonic hedgehog (Shh). Because chronic kidney disease acts as a risk multiplier in CV, Gonzalez-Calero et al. (12) analyze the role of EV in the cardio-renal syndrome, in the search for novel key targets of interaction between heart and kidneys. Studies to the role of EV in cancer are represented in this topic with two reviews and one original research article. Julich et al. (13) discuss whether the combination of EV profiling and miRNA profiling could be good biomarkers for the detection of cancer and Bruno et al. (14) review the role of EV derived from mesenchymal stem cells as therapeutic tools in tumor growth. On the other hand, the original results of Di Noto et al. (15) open new insights in EV cellular biology and in multiple myeloma therapeutic and diagnostic approaches.

In the area of regenerative medicine, EV can play a beneficial role in restoring tissue and organ damage, and may partially explain the paracrine effects observed in stem cell based therapeutic approaches. De Jong et al. (16) discuss the function and role of exosomes in regenerative medicine and elaborate on potential applications in tissue engineering.

It is our hope that the advances highlighted here and the questions raised for future consideration will generate additional discussion, drive experimental inquiry, and bring the development of EV as therapeutic tools into focus.

## Conflict of Interest Statement

The authors declare that the research was conducted in the absence of any commercial or financial relationships that could be construed as a potential conflict of interest.
